# Self-efficacy and embodiment associated with Alexander Technique lessons or with acupuncture sessions: A longitudinal qualitative sub-study within the ATLAS trial

**DOI:** 10.1016/j.ctcp.2018.03.009

**Published:** 2018-05

**Authors:** Aniela Wenham, Karl Atkin, Julia Woodman, Kathleen Ballard, Hugh MacPherson

**Affiliations:** a Department of Social Policy & Social Work, University of York, York, YO10 5DD, UK; b Department of Health Sciences, University of York, York, YO10 5DD, UK; c Society of Teachers of the Alexander Technique, Grove Business Centre, Unit W48, 560-568 High Road, London, N17 9TA, UK

**Keywords:** Alexander Technique, Acupuncture, Chronic neck pain, Participant experience, Qualitative data

## Abstract

**Background and purpose:**

A large randomised controlled trial found that the provision of either Alexander Technique lessons or acupuncture, for those with chronic neck pain, resulted in significantly increased self-efficacy when compared with usual care alone. In turn, enhanced self-efficacy was associated with significant reductions in neck pain at 6 and 12 months. In this analysis we explore the perspectives of participants within the trial, with the aim of gaining a better understanding of how these interventions had an impact.

**Methods:**

We used a longitudinal qualitative approach; in-depth interviews, informed by a topic guide, were conducted with a sample of the trial population. Participants were interviewed twice: at around six months (n = 30) and twelve months (n = 26) after trial entry. Analysis was guided by the principles of grounded theory, and key themes were developed.

**Results:**

Five key themes emerged: pre-trial experiences of biomedical treatment against which subsequent interventions were compared; emergence of tangible benefits from the interventions; factors that contributed to the observed benefits, notably growing self-care and self-efficacy; a developing sense of embodiment as an integral part of the transformative process; and contribution of these factors to sustaining benefits over the longer term.

**Conclusions:**

In-depth interviews revealed a rich array of experiences. They gave insight into the positive impact of the interventions on development of self-care, self-efficacy and embodiment. These findings complement the quantitative trial data, providing a more nuanced understanding of the factors that underpin the previously quantified improvement in self-efficacy and its association with longer-term reductions in pain.

## 1 Introduction

In a recent randomised controlled trial of Alexander Technique Lessons or Acupuncture Sessions (ATLAS), both interventions led to long-term benefits for primary care patients with chronic neck pain, with significantly reduced pain and associated disability at 12 months when compared with usual care alone [1]. For both interventions, increasing self-efficacy was found to be an important factor associated with reduced pain at both 6 and 12 months [1]. Self-efficacy was defined as the extent of the participants' confidence in their ability to reduce their pain using methods other than medication, and was assessed using a validated measure [2]. Participants attending either Alexander lessons or acupuncture sessions reported significant improvements in the way they lived and cared for themselves compared with those receiving usual care alone [3,4].

There is a growing body of evidence that Alexander Technique lessons are associated with long-term benefits, including a trial with low back pain patients showing significant reductions in pain and disability at 12 months, compared with usual care alone [5,6]. The Technique itself is a systematic approach to self-care, and a method for reducing maladaptive habit patterns through a combination of help from a teacher and the self-application of taught knowledge and skills. These are usually learnt in one-to-one practical lessons, the teacher providing integrated spoken and hands-on guidance to facilitate both cognitive and experiential learning, with the aim of improving general (mind-body) functioning [7]. Better understanding of the experiences of individuals being taught the Technique could further clarify the nature of this intervention.

Acupuncture is increasingly understood to involve longer-term benefits [8] though the mechanism for this long-term impact is less well understood. Acupuncture is commonly perceived to consist of a selection of relevant points on the body which are then needled. While this may be a key aspect of treatment, within the traditional acupuncture style, and therefore integral to the acupuncture diagnosis, another aspect is the provision of advice about life style and self-care, which are considered by practitioners as important for longer-term change [9]. Further in-depth study of the interactions and experiences of acupuncture patients will help in providing a better explanation of lifestyle and self-care advice, and potential linkage with longer-term effects.

The ATLAS trial [1] has provided a useful platform from which to explore the participants' experiences and their perspectives of potential ways in which the changes in clinical outcomes came about. In particular, we aimed to explore in depth the role of learning, self-efficacy and self-care in sustaining benefits over the longer term.

## 2 Methods

Our approach involved qualitative longitudinal research, with a prospective-repeat interview design to capture, at two time points, the experiences of participants with chronic neck pain who attended Alexander Technique lessons or acupuncture sessions. Interviews captured participants' perceptions and experiences of the interventions, and the factors that they associated with their impact over the short, medium and longer term. This distinctive methodology privileges time and context, while revealing the temporal processes in an individual's life. The contribution made to healthcare by qualitative longitudinal research has gained significant recent momentum [10], notably in relation to the treatment of long-term conditions such as cancer [11] and the management of chronic arthritis [12].

### 2.1 The context of the analysis

This qualitative study was embedded within a trial set in primary care in the UK. The trial recruited 517 patients with chronic neck pain of duration of at least three months [1]. Participants were randomised to three groups and offered up to 20 Alexander Technique lessons, or up to 12 acupuncture sessions, or usual care alone. The overall intervention time was 600 min for both the Alexander group and the acupuncture group; and all three groups continued with their usual care. On recruitment, participants were found to have had neck pain for a median of 6 years, and 60% were taking painkillers prescribed for their neck pain [1]. Outcomes were measured using the Northwick Park Pain and Disability Questionnaire [13] at baseline, three, six and 12 months. Self-efficacy was also measured at 6 and 12 months using the Chronic Pain Self-efficacy Questionnaire [2]. Additional quantitative data were collected on aspects of self-care.

### 2.2 Sampling frame

For the interviews, purposeful sampling was used to ensure sufficient diversity in gender and location across trial participants, such that potential differences could be more clearly drawn out through the analysis of the data. The purposive sampling aimed to have approximately equal numbers of those attending Alexander Technique lessons, acupuncture, or usual care alone; equal numbers of women and men; equal numbers aged under and over 50; and equal numbers across Sheffield, Manchester, Leeds, and York.

### 2.3 Data collection

Interviews were conducted at two points in time over the course of a 12-month period, the first one (Interview 1) after, or near, the end of the intervention (at 6 months) and a second one (Interview 2) at 12 months. Interviews were conducted face-to-face by the lead author, usually in the participants' own homes, and lasted between 30 and 90 min. Occasionally the participant's partner accompanied them during the interview. In Interview 1, there were 30 interviews, 7 from Sheffield, 6 from Manchester, 8 from Leeds and 9 from York. Altogether, 10 were with participants in the Alexander group, 10 with acupuncture group participants and 10 with those in the usual care alone group. In Interview 2, there were 26 interviews, 7 from Sheffield, 4 from Manchester, 7 from Leeds and 8 from York; of these, 8 were the Alexander group participants, 10 acupuncture participants and 8 usual care alone. All three randomised groups received usual care and, because of space limitations, we are reporting on data primarily from those in the Alexander and acupuncture groups. The interviews were audio recorded and transcribed verbatim.

### 2.4 Topic guide

Interviews were based on a topic guide, informed by the relevant literature and developed by the research team prior to the start of the interviews. The guides started with introductory questions regarding demographic data, the impact of neck pain and effect on life, pain management strategies, and perceptions of changes over time. Participants' experience of interventions within the trial was the next focus of questions, including prior conceptions of the interventions; what was learnt from the experience; how it impacted on pain and quality of life; what made a difference; and how the participants' views changed over time. Questions were asked about their relationship with their practitioner (acupuncturist, Alexander teacher or general practitioner). Finally we asked participants what they considered to be the most important thing learnt about how to live with neck pain.

### 2.5 Ethics

Ethical approval was granted from Leeds West Research Ethics Committee (REC ref 11/YH/0402). The trial was registered at Current Controlled Trials (ISRCTN15186354) on 14 February 2012.

### 2.6 Data analysis

After careful reading and re-reading of the interview transcripts, key themes and sub-themes were developed into a framework for organising the material in line with the conventions of good qualitative research [14]. Similarities with the principles of grounded theory were drawn upon when analysing the data [15]. This process involves the development of categories that illuminate the data, then an attempt to saturate the data in order to draw out the relevance of such categories across the wider dataset, and finally the development of key themes that can be applied to the wider social context.

## 3 Findings

Drawing on the evidence from the interviews, five key themes emerged in relation to the participants' engagement with the Alexander Technique (AT) or acupuncture (ACU): 1.) Pre-trial experiences of biomedical treatment against which the subsequent intervention was compared. This drew attention to previous medical encounters and their impact on managing chronic pain, often aligned with a process of disempowerment. 2.) Emergence of some tangible benefits, including pain reduction and movement freedom during the interventions. 3.) Factors contributing to these observed benefits, notably growing self-care, self-efficacy and a sense of empowerment, helped by a positive relationship with the practitioner. 4.) A developing sense of embodiment as an integral part of the transformative process. 5.) The contribution of all these factors to the sustaining of benefits over the longer term.

1.) Pre-trial experiences of biomedical treatment

Most participants felt their chronic neck pain had been dismissed by the medical profession. For many, medical treatment started to become part of the problem rather than the solution. Conventional forms of treatment were sometimes perceived as doing more harm than good due to the negative side effects of interventions such as painkillers. Dissatisfaction and concern with relying upon painkillers was expressed by most interviewees. Their experience of biomedical treatment over a lengthy period of time led to expressions of ambivalence and powerlessness for many:

*“I think he's [GP] just at a loss as to what to do. I've been for physio. What else can they do? I don't know. Apart from give you painkillers.” (Female, ACU, Interview 1)*

*“After you get over a certain age. They just sort of bin you. Painkillers – that's the great answer for everything.” (Male, ACU, Interview 1)*

Participants described the impact that their neck pain condition had made on their lives before entering the trial; for many this was profound. In extreme cases, feelings of desperation led to individuals having suicidal ideation:

*“I was resigned to the fact that my life was just going to be painful and I suppose at points you do consider if it's worth it. Putting up with it – life.” (Male, AT, Interview 1)*

*“I get very irritable and I can be, not nasty, but a bit snappy … because it's just constantly there and you just lash out because it's so intense sometimes. It is quite depressing … it gets me down. I do have times when I just don't want to talk to anybody.” (Female, ACU, Interview 1)*

*Everything I did it was painful … I couldn't sleep through the night cos I couldn't get comfortable … so that impacted on my daily life … it was just shameful.” (Female, AT, Interview 1)*

*“It has been difficult to deal with. It hasn't stopped me from working but it made working more difficult…. And of course, I don't sleep very well because of it, which then you're tired most of the day, which again has an impact on your working life.” (Female, ACU, Interview 1)*

The above narratives, reflecting powerlessness and elements of despair, provide a useful context in which to understand participants' initial response to the idea of different forms of healthcare; and the way they then responded to the interventions provided. Many participants described a stage in their condition where they would ‘try anything’:

*“I got to the stage where I was desperate and I thought I just want something done … because I was desperate, I accepted that I'd do it.” (Female, ACU, Interview 1)*

*“When I got the letter I was like, oh well, can't harm me, having a go.” (Female, AT, Interview 1)*

2.) Emergence of tangible benefits from the interventions

In the first wave of interviews, most participants had just completed their series of acupuncture sessions or Alexander lessons (around 6 months) and most had started to notice tangible improvements in their neck pain, as well as other benefits, including improved wellbeing. A reduction in pain was often associated with a decline in the use of painkillers, and considering interviewees' concerns surrounding the use of painkillers, this was deemed by them to be a positive outcome. Examples of emerging tangible change include:

*“The more treatments I had, the better it got … I'm walking again, I can play snooker.” (Male, ACU, Interview 1)*

*“Now I can do something about it. Got a bit more of a brighter outlook on life … Instead of just thinking, right, where's the tablets, or, there it is, it's thinking, it's there let's do something about it, relax my muscles, let's work out why it's hurting and do something about it.” (Male, AT, Interview 1)*

*“…it has helped me a lot that I haven't had to go back to the doctors and ask for painkillers. I do take painkillers now and then when I need them, but I don't ask for them all the time.” (Female, ACU, Interview 2)*

*“I've been in pain for ten years and now I would say that my pain is absolutely minimal, that today I have no pain at all, and I hadn't ever had that in the last ten years. So it's been quite a revelation.” (Female AT, Interview 1)*

Not all experiences, however, were entirely positive. In some cases, the participants felt that it just was not the right approach for them:

*“I can see where they're coming from, but almost immediately I did think to myself … I don't have time to think about anything, ever, like (laughs). It's rare that I'm sat still, so, like here. And so I was a bit like, God, how am I gonna start thinking about before I move my arm, where's my head and (laughs) what, where am I applying pressure and stuff. I just thought it's perhaps not the best for my lifestyle … it's just not suited to me … I've still had pain and I know I've created that myself probably, but it's cos I can't do what I've been taught (laughter).” (Female, AT, Interview 1)*

For some, the failure to bring about the benefits they desired or anticipated, after agreeing to the intervention, led to a greater sense of disillusionment:

*“It's one of those things that people say, oh, it's all a load of mumbo jumbo … they don't believe in it. And a part of me thinks that. And then a part of me wants it to work. Do you know what I mean … I'm desperate. I need something to work … I expected it to be a miracle cure. I wanted it to be the thing that stopped me having this pain. It hasn't had the desired effect.” (Female, ACU, Interview 2)*

3.) Factors contributing to the observed benefits

Many expressed feelings of greater control over managing their neck pain, through gaining self-awareness, skills, knowledge and changing behaviour. This was especially important for those who had previously felt disempowered when attempting to manage their condition.

*“Really uplifted by it, really empowered by it and really surprised at, at what I had experienced.” (Female, AT, Interview 1)*

*“Well with somebody else telling me sort of, it probably emphasised what I really already knew, but just in, in a position of more knowledge than I am, it sort of made me more aware that what I was perhaps doing in one direction wasn't terribly good. So she made me more aware of that really, yeah, she did … yeah, yeah she made me aware of quite a few things really.” (Female, ACU, Interview 1)*

*“You're in control, you know.” (Male, AT, Interview 2)*

*“I'm glad I took part in it, at least I know what can help, I can be a bit more aware of me triggers and try to do some thingsmeself, so. I can see some positives in it.” (Male, ACU, Interview 2)*

When reflecting on their experience of either Alexander Technique lessons or acupuncture, participants reported on some key factors that helped change to occur, leading to development of self-care and self-efficacy. For both groups, the self-awareness and skills learned from the interventions included an ability to release excessive muscular tension:

*“It's learning more about yourself and your own body and your bad habits … Because you don't realise you are doing them … So you can see a progression of it improving, because the first few actually you think, well, it's not really doing a lot, but it's getting used to putting … it's all very well doing it in the lessons but it's putting it into your everyday life so that when you go away you are still making sure that you are doing what he teaches you … If you are in pain or uncomfortable you'll bring your attention to it and kind of relax your shoulders and stuff like that … I've got a bit more control back in my life that I can manage the pain. I mean, I still have the pain, but I think it's more a way of life than an actual cure but I feel a lot better with it that I can do something about it.” (Male, AT, Interview 1)*

*“…it's (acupuncture) made me aware of limitations and trying to improve them … Before the acupuncture, when I set off to walk and I was thinking I need to straighten up here, but not know, really knowing why, and now I know my muscles are tense. So I make more of a conscious effort to relax the muscles I suppose, which I hope will help.” (Female, ACU, Interview 1)*

For those participants receiving acupuncture, ability to change often stemmed from advice about diet and exercise:

*“…to try and keep doing the exercises. Again, like (the acupuncturist) said, she gave us a load to do and try keep with them and, to keep it free and that … sort of like, you know, doing the exercises properly, more, more, you know, properly and that like.” And then at the 12 month interview: “As long as I keep doing the exercises I think and keep things moving I, yeah I think it (the neck pain) will (be gone forever), and not being stupid like, you know, you can do, be stupid and pull muscles and, and things, and do things like that, which can set it off.”(Male, ACU, Interviews 1 and 2)*

*“Yes, she told me to relax more if I can and she gave me some exercises to do, light exercises to do…. she gave me an instruction sheet for the next lesson to do, things like that … she said to me loads of vegetable which she knows I do. Plenty of fruit, chicken, fish, things like that which she knows I do anyway. She said, well, if you do that anyway.” (Female, ACU, Interview 1)*

*“Yeah, keeping it, just keeping your head mobile yourself really, and if you can do that then that's great, cos I mean if you don't then obviously it would stiffen up and then you wouldn't be able to do anything.” (Female, ACU, Interview 2)*

For those attending Alexander lessons, greater self-efficacy and self-care began with becoming more self-aware and learning how to apply the Alexander thinking skills:

*“with this you don't really have to physically do anything, you've just got to think it, so it's all in the head … So you can be walking down the street and you can put it into practice, I can be at work … I had made my muscles go soft that for ten years hadn't been, and that was just from my teacher just explaining what to do and just very lightly touching my shoulders and just, I don't know, just talking me through it.” (Female AT, Interview 1)*

*“I couldn't believe that just something so simple and so easy to do, once you know sort of how to do it, and even at a time when I was actually seeing the benefits, I still couldn't believe that just sort of thinking about walking taller and thinking every time you get out of a chair, and I, I couldn't believe, even though it was working, that it was actually that that was working, you know.” (Female AT, Interview 1)*

*“…because I've learnt a technique and because I keep using it, I don't think I'm gonna forget it, even though I'm obviously not having as much, or near as much neck pain, I can't see that anything's gonna get worse now.” (Female, AT, Interview 1)*

Interviewees discussed the positive, therapeutic relationship, which had developed with Alexander teachers and acupuncturists. Having the opportunity and time to develop rapport was seen as significantly different from the situation of their GP, who was sometimes perceived to lack time and empathy. Many participants also felt their GP lacked an understanding of their condition.

*“It's good that someone's actually taking it seriously … it's been great, she actually spends some time and listens to what I've got to say, you know, get a full history of it and, you know, and make, and make a note herself how I'm progressing with it and things like that … I trust her.” (Male, ACU, Interview 1)*

*“I think we work really well as a team. And I think because I went in with open eyes, she was very much prepared to, to work with me to whatever level she could.” (Female, AT, Interview 1)*

*“I felt a bit more optimistic that somebody was trying to help me and therefore, maybe I was, should help myself a bit more.” (Female, ACU, Interview 1)*

A few participants expressed initial feelings of vulnerability that were only dispelled once a relationship was established with their practitioner. The use of touch (albeit professionally trained) with both these interventions and a gender difference with the practitioner were at the root of such anxieties:

*“When I got there I felt quite vulnerable … I just had no clue what to expect … he wasn't touching me inappropriately … but when you're at a GP and they put your hands on you, it's different because you know they're your GP … maybe I had felt a little different had it been a woman … obviously he's got to know me and I've got to know him, which is much better. We've developed a rapport which is great.” (Female, AT, Interview 1)*

*“I think you have to be very confident and comfortable with a person because …. when you are lying there with your bra undone and just your knickers or your trousers on with a load of needles in your back … you are very, very vulnerable, you know?… But she never made me feel embarrassed or what have you. So yes, she was genuinely a nice lady…. you have to be open to the person who is giving you the treatment as well, because I would imagine if you don't like them, again, you are in a vulnerable position…. I wouldn't be comfortable with a chap doing it, no.” (Female, ACU, Interview 1)*

4.) Embodiment integral to the transformative process

Some participants described a re-evaluation of their identity, for example, one individual expressed the feeling of being a ‘new person’. For many, the experience of increased self-awareness and a sense of interconnectedness and embodiment were integral to the transformative process. The perception of ‘neck pain’ could no longer be reducible to a ‘body part’:

*“She looked at me as [a] whole rather than as a shoulder and a neck … And I'm not just learning to relax certain muscles that were the problem, it was everything, which, I suppose in some respects, just balanced, balanced me a lot better.” (Female, AT, Interview 2)*

*“She's on about points in the body, you know, muscle all connected … so that's, it's things like that where you think ah there's a little bit of something where everything else is linked in … I mean it's like she stuck (a needle) up on me neck up there and I could feel it down here just like relaxing, like nerve, just like a ripple down, I felt that was, that, you know, nice, you know, but yeah.” (Male, ACU, Interview 1)*

For some, questioning mind/body dualism was a critical feature of this shift, with the realisation of the inseparable links between physical, emotional and social attributes, including pain:

*“I think I've got a little bit more self-esteem, because … even though it's not a posture thing, you do feel as though you're in a better position when you're standing or when you're sitting or whatever, and I think that makes me feel a little bit taller, it makes me feel a little bit more elegant, it makes me just feel a little bit nicer about myself.” (Female, AT, Interview 1)*

*“It relaxed, it relaxed the muscles and made them freer and it was easier really, and I didn't, I wasn't conscious of having stiffness … I mean acupuncture is all really about being relaxed and that obviously helps your body. Tensing, you're fighting, you're fighting what you're trying to overcome.” (Female, ACU, Interview 1)*

Some participants expressed how their greater self-awareness and sense of self, gained through the experience of Alexander Technique lessons or acupuncture, was crucial to enabling behavioural change:

*“It was weird cos I felt like I was learning how to walk again (laughs) sometimes, because you do pick up habits as you get older don't you? You take more notice of things you're doing, like lifting. We take it all in our stride, don't we, day-to-day, we do all these things without even thinking about it. She teaches yah to think about things …. she's taught me to think of how I'm moving and walking, even getting off a couch, from being laid down and things like that, and it all just starts to flow. It's natural once you've learnt it.” (Female, AT, Interview 1)*

*“But one thing I did discover with the acupuncture is my back muscles at the top are quite tense, and possibly I'm tensing myself against that sort of thing, well obviously I suppose I am, you know.” (Female, ACU Interview 1)*

*“I'm a much calmer person, it's taught me how to take a step back and assess a situation rather than jump straight in … because I've learnt how to do it, I've learnt how to take a step back, I've learnt how to relax my body.” (Female, AT, Interview 1)*

*“…while having the acupuncture I discovered that, what I'm aware of now is when I start walking I'm conscious, I have to make a conscious effort to straighten up, while I was having the acupuncture it seemed to happen on its own, I seemed to be straighter, I did seem to be straighter, and the movement was better…” (Female, ACU, Interview 1)*

5.) Sustaining benefit over time

During the second set of interviews at 12 months, participants described how they continued to use the understanding and skills they had gained, after the interventions had ceased, to sustain and in some cases further improve their reduction in neck pain:

*“I've managed to keep it at bay. Yeah, sometimes if say I've slept in a funny position I'll wake up and think of, neck feels a little bit off today. But then I sort of take extra care of how I'm sitting and do what I've been taught and it's just, I can clear it sort of with a couple of paracetamols, if I need to, and sometimes I don't need to take anything at all.” (Female, AT, Interview 2)*

*“So I think, you know, just things in moderation and doing the exercises that she gave me to do, I think it'll sort of keep it long-term” (and his wife adds): “I think also it's made yer sort of rethink, cos originally you were planning on retiring at, at sixty-three because of all the aches and pains, weren't it, but now he's decided that he'll probably stay till he's sixty-five.” (Male, ACU Interview 2)*

*”The positive thing about (the Alexander Technique) is you can carry on doing the things that the teacher's taught yah to help yah, and I do. And gradually it's just got better and better, you know. And as for life changing, probably the Alexander's changed me because I never used to realise it, but with being in pain you used to tend to be a bit short tempered and…. grumpy.” (Male, AT, Interview 2)*

*“I'm virtually pain free at the moment … now whether that is due to a certain degree (of) lifestyle change, because I'm doing a lot more exercise, dieting, been on holiday for the first time in ten years, so.” (Female, ACU, Interview 2)*

## 4 Discussion

Our findings highlight several key features in the process of change for trial participants with chronic neck pain attending either Alexander Technique lessons or acupuncture sessions. We found that many people are willing to embrace change when they are helped by an appropriately trained professional with whom they can form a supportive relationship. The contrasting experiences of treatment received in primary care provided a backdrop for comparison. Chronic neck pain is perceived differently by different individuals, and trial participants' experiences of Alexander Technique lessons and acupuncture also varied. However, most participants experienced tangible benefits, including pain reduction and increased feelings of well-being and empowerment. A growing awareness of excessive muscle tension and the ability to release it (to varying extents) and thereby gain some pain relief, was mentioned by participants in both groups. Particular strategies for avoiding pain were also reported to be of value in sustaining longer term benefits.

Participants also acknowledged behavioural changes they themselves had made and which formed a key part of the better self-care that led to the on-going improvement in wellbeing and pain reduction. Another key finding was that increasing self-awareness allowed some participants to recognise that changes were becoming embedded at both physical and mental levels, leading to a sense of embodiment that contradicted conventional ideas of mind/body dualism.

A strength of this study is that we followed a well-constructed protocol to recruit a diverse range of participants from within a randomised controlled trial using a sampling frame. The participants were typical of the many individuals in primary care who continue to have considerable neck pain, despite access to medication and other forms of usual care such as physiotherapy. The interviews were all recorded and transcribed verbatim, providing a strong basis for data analysis. A constraint on data collection was the relatively fixed time point for interviews, for Interview 1 at approximately 6 months and at follow-up for Interview 2 at 12 months. With only 10 participants in each of the Alexander and acupuncture arms, we may have had too few participants to capture additional key factors. Although we have reported on the participants' experiences of usual care in the acupuncture and Alexander groups, we have not reported on the experiences of those allocated to the usual care arm of the trial because of limitations of space.

Our results are consistent with the findings of other studies of the Alexander Technique, including our sister papers, that provided quantitative data on the importance of self-efficacy for greater reductions in neck pain [1,4]. Interviews with participants who had attended Alexander lessons in the back pain clinical trial, as well as those in a pain clinic setting, revealed similar experiences of increased self-efficacy in managing pain, and feelings of gaining more control in general [16,17]. Furthermore, a small pilot study of patients with arthritic knee pain, reported a direct relationship between pain intensity and excessive forces caused by maladaptive (habitual) muscular co-contraction, with both decreasing significantly following Alexander lessons [18]. Findings across these different studies illustrate how learning the Alexander Technique enabled individuals to improve the way they live their lives and manage their everyday activities through increased awareness and purposeful direction of thought.

There is also some consistency within the literature on acupuncture, specifically with the quantitative data on acupuncture and self-efficacy [1,3] and a qualitative study on acupuncture for low back pain that reports on life-style and self-care changes recommended by practitioners [9]. The latter study identifies these components of acupuncture as “specific” to the intervention, because they are based on the theoretical framework that underpins a traditional acupuncture diagnosis. In another qualitative study involving audio recordings of interactions between acupuncturist and patient within the traditional acupuncture consultation, self-care was found to be integral, interactive, and individualized [19].

Our findings illustrate the diversity of the process of change that can result from Alexander lessons or acupuncture sessions for individuals with chronic neck pain. Some of this diversity reflects the differing balance of therapeutic and educational elements between the two interventions. As previously reported, acupuncture can be seen as predominantly therapeutic but with an important educational component, primarily through lifestyle advice such as exercise and diet [3]. In contrast, while Alexander Technique lessons also have a therapeutic element, they are primarily principle-based education in the use of embodied thinking skills that enable people to improve muscle tone, postural support, movement coordination and balance, so reducing maladaptive musculoskeletal habits and the pain they cause [4].

Further research into Alexander Technique lessons or acupuncture could benefit from the introduction of a more nuanced approach to outcomes evaluation, for example by exploring factors that may enhance or be unhelpful to a positive outcome. Of particular interest would be exploration of the raising of self-awareness and sense of embodiment, and the extent to which acceptance of mind-body unity might be an essential prerequisite to beneficial change. Research on how to best engage individuals and help them optimise their learning would also be of interest and value. In terms of practice, our findings reinforce the importance of recognising the processes that facilitate the development of self-care and self-efficacy. Our evidence supports the contention that the environment in which these processes flourish is an integral part of both interventions.

Our qualitative findings contribute to a better understanding of the observed quantitative results from the ATLAS trial. We have shown how participants' experiences gained from Alexander lessons or acupuncture sessions enabled behavioural change towards better self-care and led to a sense of embodiment. These changes underpin the observed quantified improvement in self-efficacy and the associated longer-term reduction in pain.

## Conflicts of interest

HM reports that he is a member of the British Acupuncture Council. JW and KB report that they are members of the Society of Teachers of the Alexander Technique.

## Contributors

AW, KA, JW, KB and HM designed the study, AW collected the data and conducted the first analysis of the data, all authors contributed to subsequent analyses and the writing up of the study, and agreeing the final version of the manuscript.

## Financial support

The ATLAS trial was sponsored by the University of York. This research was funded by a clinical studies grant No. 19702 from Arthritis Research UK.

## Appendix A Supplementary data

Data profile Data profile
